# Identifying individuals at clinical high risk for psychosis using a battery of tasks sensitive to symptom mechanisms

**DOI:** 10.21203/rs.3.rs-5005564/v1

**Published:** 2025-05-08

**Authors:** Trevor Williams, Jim Gold, James Waltz, Jason Schiffman, Lauren Ellman, Gregory P. Strauss, Elaine Walker, Scott W. Woods, Al Powers, Joshua Kenney, Minerva Pappu, Philip Corlett, Tanya Tran, Steven Silverstein, Richard Zinbarg, Vijay Mittal

**Affiliations:** Kent State University; MPRC; Maryland Psychiatric Research Center; University of Georiga; Emory University; Yale University School of Medicine; Yale University School of Medicine; Yale University; University of Rochester Medical Center; University of Rochester Medical Center; Northwestern University

## Abstract

The clinical high risk for psychosis (CHR-P) population is important for understanding disease progression and treatment; however, standard approaches to identifying CHR-P individuals are expensive and labor-intensive. Focusing on neurocognitive mechanisms that underlie individual psychosis symptoms (positive, negative, and disorganization) may improve screening and identification. The present study examines whether a behavioral task battery that assays symptom mechanisms can identify CHR-P individuals and predict risk severity. Participants (*N* = 621) were recruited from clinics and the community as part of the Computerized Assessment of Psychosis Risk (CAPR) consortium study. Structured clinical interviews, a dimensional risk calculator, and behavioral tasks were administered. Clinical interviews identified the following groups: (a) CHR-P (*n* = 273), (b) non-CHR-P individuals with limited psychosis like experiences (PLEs; *n* = 120), (c) participants with mental disorders and no PLEs (CLN; *n* = 82), and (d) healthy controls (HC; *n* = 146). Multinomial logistic regression indicated that the task battery differentiated groups (*p* < .001), with utility for identifying CHR-P individuals (Sensitivity = .87, PPV = .51, NPV = .77), though with high false positives that varied based on comparison group (Specificity = .21-.43). Tasks also predicted psychosis risk calculator scores (Adjusted *R*^*2*^ = .12), with the two unique predictors being positive symptom task variables associated with updating beliefs regarding environmental volatility. Overall, symptom mechanism tasks differentiated CHR-P individuals from control groups, suggesting their potential as novel screening tools. Using tasks to more efficiently identify CHR-P individuals (e.g., enrich samples), may lower barriers and identify individuals that may otherwise be missed.

## Introduction

Psychosis spectrum disorders (e.g., schizophrenia) are a major source of disability worldwide and cause considerable personal suffering [1]. Recently, increased emphasis has been placed on early identification, prevention, and mechanism research, in hopes of better understanding and mitigating the impacts of these disorders [2]. Research on individuals at clinical high risk for psychosis (CHR-P) has been at the forefront of psychosis risk research; however, these individuals are challenging to identify. Complicating matters, other clinical populations have overlapping symptoms and some individuals report psychosis-like experiences (PLEs) of limited frequency, intensity, and impact, such that they are not considered to have “at-risk” status. The present study tests a novel approach to identifying CHR-P individuals: a behavioral task battery that assesses underlying symptom mechanisms.

CHR-P individuals experience attenuated positive symptoms of psychosis (e.g., seeing human-like shadows), brief full positive symptoms, or genetic risk for psychosis combined with functional decline [2]. A recent meta-analysis estimated that 25% of these individuals later develop a psychosis spectrum disorder [3] and CHR-P individuals who do not often still experience significant symptoms and impairment [4]. Given that these participants often are unmedicated and have not developed full symptomatology, researching and intervening on CHR-P may advance our understanding of symptom progression, decrease duration of untreated psychosis, and provide a window for preventive treatments [2].

One challenge to CHR-P research is that it is resource-intensive and costly to recruit participants. Structured clinical interviews are the gold-standard approach to CHR-P identification, but take 1.5–2 hours to administer and require considerable training [5]. Self-report questionnaires are another screening tool, but are limited in this population by poor insight and guardedness [6]. The present study builds upon advances in clinical cognitive neuroscience and computational psychiatry to consider a new approach: behavioral tasks focused on symptom-specific mechanisms.

Across the psychosis spectrum, progress has been made identifying cognitive mechanisms that underlie specific symptoms [7]. This literature is defined by its emphasis on individual symptoms, over diagnostic constructs, and integrating sophisticated behavioral tasks with neural markers. For instance, research on delusions has indicated that aberrant prediction error signaling may lead to the development of spurious beliefs or a sense of unpredictability, which form the seeds of delusions [8, 9]. Similarly, hallucination research suggests that the overweighting of prior beliefs (e.g., perceptual expectations) may influence the processing of sensory information, such that aberrant patterns are formed [10]. Negative symptoms have been connected to reward processing and response initiation, observed in how individuals with negative symptoms represent and update beliefs about expected rewards and the degree of effort required to obtain them [11, 12]. Finally, disorganization symptoms have been linked to reductions in context processing, perceptual organization, and motor coordination [13].

Symptom mechanism tasks have considerable promise for identifying CHR-P individuals, in that they capture the insights of neuroscience research, without requiring resource-intensive neural data. Despite this, these tasks have yet to impact CHR-P identification for several reasons. First, symptom-specific tasks have often been studied in isolation to each other, limiting their ability to identify CHR-P individuals, who experience varied symptoms. It is necessary to examine their collective predictive power and potential as a testing battery. A second limitation, which extends beyond behavioral task research, is the limited consideration of clinical controls [14]. Although demonstrating the ability of a measure to differentiate CHR-P from healthy individuals is useful for community screening, the reality is that much screening is done in help-seeking populations [15]. A stronger test of CHR-P identification would thus involve comparisons to groups with varied clinical symptoms and especially to individuals with PLEs that fall short of meeting CHR-P criteria.

The present study represents the first step in a broader program of research aiming to fundamentally reshape psychosis risk research, using symptom-specific mechanisms [16]. Specifically, this study examined behavioral tasks that assess symptom mechanisms as predictors of CHR-P status in a large sample. Notably, this included not only differentiating CHR-P individuals from healthy controls, but also from individuals with lower-risk PLEs and other clinical diagnoses. It was hypothesized that the task battery would significantly differentiate CHR-P individuals from all three groups; however, because these tasks are so often studied individually and without clinical controls, specific predictions were not made regarding which tasks would be predictive. Finally, relations to a conversion risk calculator were examined within CHR-P individuals, as such risk calculators are predictive of development of a psychosis spectrum disorder in this population and have recently been used to enrich CHR-P samples [17].

## Methods

Data were baseline assessments from the Computerized Assessment of Psychosis Risk (CAPR) study, a multisite study of psychosis risk (see published protocol, [16]). Participants (*N*=621) completed assessments between January 2020 and July 2023, which included clinical interviews, questionnaires, and a behavioral tasks. CHR-P (*n*=273) participants were included based on meeting criteria for a psychosis risk syndrome. Three control groups were included, consisting of individuals experiencing psychosis like experiences (PLEs) that did not meet CHR-P criteria (PLE group; *n*=120), individuals without PLEs but with other current or recent clinical diagnoses (e.g., Major Depressive Disorder; CLN group; *n*=82), and healthy controls without PLEs or psychopathology symptoms (HC group; *n*=146). Exclusion criteria for all participants were severe head injury, the presence of a neurological disorder, and lifetime history of a psychotic disorder. The study was approved by the Northwestern University IRB (STU00211351) and acknowledged by the IRBs of participating sites. All adult participants provided informed consent after receiving a complete description of the study, whereas minors provided written assent and their parents or guardians provided written consent.

Interviews were conducted by formally trained postdoctoral fellows, graduate students, and research assistants. Behavioral tasks were administered online by research assistants trained to explain task instructions and monitor participant performance for data quality. Participants self-reported on demographic characteristics in a format consistent with the United States National Institutes of Health reporting requirements.

### Clinical Measures

*The Structured Interview of Psychosis Risk Symptoms (SIPS)* is a semi-structured interview that identifies psychosis risk syndromes [5]. Positive symptoms are central in identifying psychosis risk syndromes and include unusual/delusional thoughts, suspiciousness/persecutory ideas, grandiosity, perceptual abnormalities, and disorganized communication.

*The Structured Clinical Interview for DSM-5, Research Version (SCID)* [18] was used to determine the presence of other mental disorders and thus inclusion status for CLN and HC groups. Interviewers administered psychotic, bipolar, depressive, anxiety, obsessive-compulsive, trauma-related, eating, and substance use disorder modules.

*The North American Prodrome Longitudinal Study-Risk Calculator (NAPLS-RC)* [19] was developed in a large longitudinal study of CHR-P individuals to predict the probability of transitioning to a psychosis spectrum disorder during a 2-year timespan. The NAPLS-RC uses SIPS ratings and participant age, as well as several other measures that were administered as part of the CAPR study: the Brief Assessment of Cognition in Schizophrenia [20], the Hopkins Verbal Learning Test-Revised [21], negative life events scores from the Research Interview Life Events Scales [22], and social functioning decline as measured by the Global Functioning Scale [23]. In the present study, it was used as a dimensional assessment of psychosis risk within CHR-P individuals.

### Behavioral Tasks

Eleven behavioral tasks were administered by trained research personnel that monitored task engagement and performance. They were presented in randomized order, with each contributing one or two predictor variables to analyses. These tasks and indices are briefly reviewed here; more information can be found in the CAPR protocol paper and the supplement to this article [16]. Descriptive statistics and correlations with symptoms are provided the supplement to this article.

#### Positive Symptom Mechanisms.

The *Kamin Blocking (KB)* task assesses aberrant causal learning and produces scores for overall causal learning (control) and inappropriate learning of “blocked” cues (blocking) [8]. The *Probabilistic Reversal Learning (PRL)* task measures prior beliefs and learning about environmental volatility, with two variables: lose-stay rate (LSR) and win-switch rates (WSR) [9]. The *Sine Wave Speech (SWS)* task assesses influence of priors on auditory perception, with the main variables being increases in sensitivity (SWS *d’*) across the task and early bias (SWS c) [24]. The *Conditioned Hallucinations (CH)* task measures prior influence (v) on perceptual judgments about the presence of an auditory stimulus presented concurrently with a visual stimulus; the probability of the target auditory stimulus changes over time and the participant’s learning about this change is captured by the volatility belief evolution rate ( _3_) [10].

#### Negative Symptom Mechanisms.

The *Gain vs. Loss Avoidance Task (GLIAT)* task measures expected value estimation through two indices that capture enhanced learning about losses relative to rewards (learning, transfer) [11]. The *Effort Expenditure for Rewards (EEFRT)* task assesses over-estimation of effort costs through the tendency to choose high effort tasks that are coupled with high reward probability (EEFRT hard) [25]. The *Delay Discounting (DD)* task measures the valuation of future rewards, with the central outcome being large reward trial performance (DD large) [12]. The Hedonic Reactivity (HR) [26] task measures hedonic response to pleasant visual stimuli (HR positive). The *Computerized Finger Tapping (CFT)* task assesses initiation and persistence of volitional movements, with average number of taps in a speeded condition being the central outcome (CFT speed) [27].

#### Disorganization Symptom Mechanisms.

The *Ebbinghaus IIlusion (EI)* task measures susceptibility to the classic illusion, producing a context sensitivity score [28]. The *Mooney Faces (MF)* task involves integrating degraded visual information into face percepts, both when faces are upright and inverted, producing separate scores for each condition [29].

### Statistical Analyses

Analyses were conducted in R [30] and the *mice* package was used for multiple imputation of missing data, with results pooled across 100 imputed datasets [31]. Multinomial logistic regression was used to predict group status, with CHR-P used as a reference group contrasted with each control group. This analysis produced three separate equations and coefficients for each predictor, such that they indicated differences between the reference group (CHR-P) and *one* of the control groups. An intercept model was estimated as a baseline, followed by adding potential demographic covariates (age, sex, race, and household income). Following this, a model with all task indices was tested. Model comparisons were made using the Akaike Information Criterion (AIC; lower is better) and the Wald test for model differences (*p<*.05). All *p* values reflect two-tailed tests.

The classification value of models was further examined through calculating sensitivity, specificity, positive predictive value (PPV), and negative predictive value (NPV) for identifying CHR-P individuals. The task battery’s ability to predict risk severity within CHR-P individuals was examined through a multiple regression analysis in which the NAPLS-RC was the dependent variable and task variables were entered as predictors.

## Results

Participants (*N*=621) were on average 23.59 years old (*SD*=4.15), and racially diverse (see Table 1). There were no large differences between groups on demographic variables, though there was a small sex difference (*p*=.04). Most participants (55%) had no missing data and the average missingness across tasks varied (12%–22%; see Table 2 descriptives). There were few extreme outliers across tasks (+/− 5SD) and these values were marked missing. Correlations between tasks were small (*M =*.01, *SD*=.10), with the exception of the correlation between the inverted and upright Mooney Face task variables (*r*=.68, *p<*.001), suggesting the tasks provide information on unique and distinct mechanisms.

Multinomial logistic regression models were fit to examine the value of behavioral tasks for differentiating groups (see Table 3). An intercept model and one with demographic control variables were compared; the Wald test (*p=*.39) and AIC (Intercept, 1604 vs. Demographics, 1620) did not indicate differences, suggesting demographic variables did not differentiate groups. Next, the intercept model was compared to one with all tasks entered as predictors. Both the significant Wald test (*p<*.001) and difference in model AIC indicated that the task battery fit better than the intercept model. Further emphasizing the predictive value of the task battery, classification accuracy statistics indicated that the model correctly identified most CHR-P individuals (Sensitivity=.87) and that a model prediction of “not CHR-P” was relatively accurate (NPV=.77); the model had somewhat low specificity (.33) and thus a propensity toward false positives, lowering the accuracy of model predicted CHR-P status (PPV=.51).

**Figure 1:**
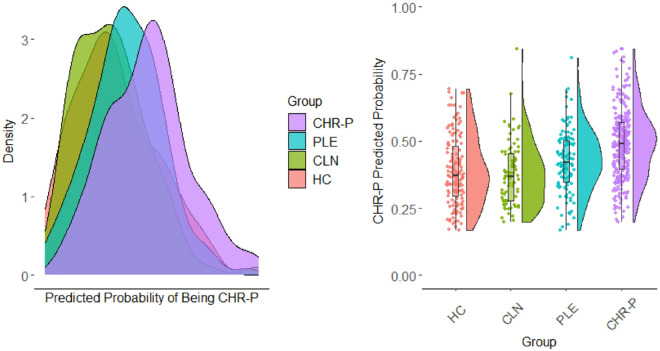
Distribution of predicted probability of CHR-P status by group. CHR-P = clinical high risk for psychosis, PLE = psychosis like experiences that are not CHR-P, CLN = clinical disorders without PLEs, and HC = healthy controls.

Next, the individual predictive equations that contrasted CHR-P and specific control groups were examined in terms of predicted probabilities (see Figure 1), classification accuracy, and significant predictors (see Table 4). The model performed best at separating the CLN group from CHR-P individuals (e.g., PPV=.84), driven by a relatively lower rate of false positives in this group (Specificity=.43). The MF inverted condition variable was the only predictor that significantly (OR=0.49; *p<*.001) differentiated groups, with the effect opposite of what is found in schizophrenia, but consistent with a recent CHR-P study [29]; a model in which only this variable was entered did not change this result (OR=0.57, *p<*.001), suggesting the effect was not driven by suppression. The model performed nearly as well at differentiating HC from CHR-P individuals (e.g., Specificity=.38, PPV=.72); however, there were a larger number of significant predictors (*p<*.05), which spanned symptom domains. Notably, the contribution of MF inverted paralleled the CLN model; however, there was also an effect in the opposite direction for KB control, which did appear to be a suppression effect (single predictor model OR=0.86, *p=*.16). Finally, although the model still was accurate in identifying CHR-P individuals relative to the PLE group (Sensitivity=.87, PPV=.71), many PLE individuals were incorrectly identified as CHR-P (i.e., elevated false-positives; Specificity=.21, NPV=.42). MF inverted was again a significant predictor and El context was a significant predictor, but similar to MF inverted it was predictive in a direction counter to theory and remained so when examined as a single predictor (e.g., OR=0.77, *p*=.024)

Finally, the task battery’s ability to predict probabilistic risk for developing a psychotic disorder (NAPLS-RC) was examined using linear multiple regression^1^. As is standard, the NAPLS-RC was examined only within CHR-P individuals (n=273) and the adjusted *R*^*2*^ for this model was .12 (95% Cl = [.04, .22]), though a similar result also emerged in the full sample (adjusted *R*^*2*^=.10; see Supplement). Overall, the task battery was substantially related to psychosis risk, with two unique predictors emerging: PRL WSR (β=.17, p=.023) and CH ^3^ (β=.14,p=.045).

## Discussion

One challenge to psychosis risk research is identifying at-risk individuals, especially among help-seeking populations. The present study took a novel approach to this problem, examining whether symptom mechanism tasks can differentiate CHR-P individuals from control groups that mirror real-world recruitment populations. The task battery successfully classified CHR-P individuals and predicted risk severity, suggesting that this approach may complement existing methods through its distinct advantages.

The sensitivity of the task battery for identifying CHR-P was high (i.e., 87%); however, the battery’s predictive accuracy (i.e., PPV, NPV) varied. The task battery was most accurate for differentiating CHR-P individuals from individuals with psychopathology and healthy individuals, but had more false positives and less accuracy in comparisons with individuals with PLEs. The sensitivity of the task battery minimally suggests utility for screening or “enriching” samples, reducing interviewing time and allowing collection of larger samples with high proportions of CHR-P individuals. Consistent with this, the battery’s relation to the NAPLS-RC within CHR-P individuals suggests the task battery predicts risk severity. Nonetheless, the false positive rates indicated that interviewing is still needed to definitively identify cases, particularly when differentiating individuals with low risk PLEs (e.g., low frequency) from individuals that are truly at-risk. Notably, these findings compare favorably to studies using neuroimaging indicators to predict psychosis risk (e.g., 51% PPV) [32, 33].

Beyond demonstrating a task battery’s potential utility, the present study may suggest how to optimize task batteries, through specific predictors. First, the Mooney Faces inverted variable was the strongest predictor of CHR-P status^2^; however, disorganization mechanisms did not drive this effect. The Mooney Faces task requires individuals to detect faces in a visually degraded picture and, although challenging for individuals with schizophrenia, it may be that CHR-P individuals engage compensatory processes that over-integrate stimuli. The present finding is consistent with a recent CHR-P study [29] and may parallel the predictive coding mechanisms underlying positive symptoms [8, 24]. Consistent with this, positive symptom mechanism tasks also identified CHR-P individuals and the two predictors of risk severity (NAPLS-RC) were from positive symptom mechanism tasks. In particular, the variables related to the NAPLS-RC, captured beliefs about changing learning environments, with one reflecting perceived environmental volatility relevant to paranoia (PRL WSR) [9] and the other a failure to update perceptual beliefs as contingencies change (CH _3_; e.g., persisting conditioned hallucinations) [10]. Belief updating has previously distinguished patients with schizophrenia from non-clinical voice-hearing controls [10], suggesting that these processes may represent important mechanisms underlying conversion to psychosis. Despite the emphasis on positive symptoms in the present findings, one negative symptom task showed relevance for predicting CHR-P status (CFT), suggesting value in continuing to explore other symptom mechanisms for identifying CHR-P individuals.

In considering the value of a task battery, it is worth contrasting it with existing methods. Focusing on the significant predictors of CHR-P status, tasks took 8–10 minutes to complete [16] and administering *all* such tasks would take 56 minutes, though further task and battery refinement may reduce administration time. Minimally trained research assistants administered tasks online; however, these tasks have also been successfully administered without supervision, suggesting that these tasks may offer a scalable and low-burden approach to screening [9]. In contrast, the SIPS and similar interviews take 1.5–2 hours to administer [34], require intensive training, and ongoing clinical supervision. Although self-report measures provide another brief screening option, they require insight and self-disclosure that may not be present in this population. Both interviews and self-reports may also be affected by symptom associated stigma, which limits disclosure [35, 36], and cultural variation in how symptoms are described [37, 38]. In contrast, behavioral tasks do not require insight or self-disclosure, and are less directly mediated by language. As such, symptom mechanism tasks provide an intriguing alternative to screening and identifying CHR-P individuals, representing a potential practical application of computational psychiatry [39, 40].

### Limitations and Future Directions

There are several limitations to this study worth considering. First, the specificity of the battery for identifying CHR-P individuals was relatively low in the full sample and in particular when individuals with PLEs were considered. Nonetheless, performance was stronger in controls without PLEs and the current battery may still be useful as a pre-screening tool for sample enrichment and future work may refine the battery, improving specificity. For instance, each task had additional indices and many would be amenable to computational modeling, which may ultimately enhance prediction [9, 16]. Future work should optimize individual tasks for efficiently identifying CHR-P individuals. Second, although this study demonstrates the value of symptom mechanism tasks for screening and identifying psychosis risk, it does not present a ready-to-use tool. Further work is needed to hone a task battery and develop implementation infrastructure. Finally, the present study focused on cross-sectional data and future work would do well to include longitudinal data and outcomes (e.g., conversion to psychosis).

## Conclusions

This study demonstrated the potential value of symptom mechanism tasks for identifying CHR-P individuals and assessing psychosis risk severity, with comparisons to control groups of varied composition. Positive symptom mechanisms were most effective for identifying CHR-P individuals. They were particularly accurate at differentiating CHR-P from individuals with other mental disorders and healthy individuals, but had higher false-positive rates when differentiating CHR-P from individuals with low-risk PLEs (less frequent, intense, etc.). Overall, these symptom mechanism tasks may have value for screening, oversampling, and identifying psychosis risk, without the resource costs of clinical interview or subjectivity of self-reports.

## Figures and Tables

**Table 1.** Demographics by clinical group

	CHR-P	PLE	CLN	HC
*N*	273	120	82	146
*M* Age (*SD*)	23.40 (4.30)	23.70 (4.35)	23.95 (3.59)	23.58 (4.06)
Sex				
Female	67.30%	66.95%	61.73%	53.79%
Male	32.70%	33.05%	38.27%	46.21%
Race				
African American	17.05%	16.95%	13.58%	13.79%
American Indian^1^	0.76%	0.69%	0.00%	1.69%
Asian	17.80%	22.03%	22.22%	31.72%
Caucasian	51.52%	50.00%	53.09%	44.83%
Multiracial	10.61%	6.78%	9.88%	8.28%
Native Hawaiian or Other Pacific Islander^1^	0.76%	0.69%	0.00%	0.00%
Unknown or not reported^1^	1.52%	0.00%	1.23%	2.54%
Hispanic	14.45%	11.02%	12.35%	7.59%
*Mdn* Household Income	$60,000.00	$60,000.00	$95,500.00	$100,000.00
Current Medication				
Antidepressant	23.00%	24.00%	17.00%	8.60%
Antipsychotic	6.50%	0.91%	0.00%	0.00%
Mood stabilizer	6.20%	1.80%	1.30%	0.00%
Stimulant	6.20%	2.70%	1.30%	0.74%
Anxiolytic	7.30%	5.50%	4.00%	1.50%
Current Substance Use Disorder^2^	30.33%	21.98%	17.91%	00.00%

*Note.* CHR-P = clinical high risk for psychosis, PLE = psychosis like experiences (not meeting CHR-P criteria), CLN = current or recent mental health diagnoses, and HC = healthy controls.

1 = due to low prevalence, these groups were combined for analyses. Only a small significant difference between groups was present for sex (*p* = .04) and, as noted below, when demographic variables were considered as a whole there were no differences between groups.

2 = Based on the presence of any current SCID Substance Use Disorder.

**Table 2.** Descriptive statistics for continuous predictors and outcomes

	N	M	SD	Mdn	Min	Max	Skew	Kurtosis
NAPLS-RC	205	12.15	7.52	10.29	3.19	41.09	1.65	2.51
KB Blocking	484	−0.05	0.74	−0.18	−1.00	1.00	0.18	−1.48
KB Control	483	0.59	0.57	0.84	−1.00	1.00	−1.69	1.85
PRL WSR	524	−1.25	0.55	−1.29	−2.00	−0.01	0.26	−1.01
PRLLSR	524	0.24	0.21	0.18	0.00	1.00	0.82	−0.22
SWS d’	521	−0.08	0.86	−0.04	−2.46	2.69	0.02	0.18
SWS c	521	−0.29	0.62	−0.28	−2.01	2.01	0.14	0.34
CH v(prior)	495	0.66	0.62	0.52	0.12	3.47	2.47	6.46
CH _3_	493	−6.01	0.47	−6.00	−8.16	−3.54	0.70	10.95
HR positive	518	3.94	0.71	4.00	1.00	5.00	−1.04	1.54
EEFRT hard	514	0.55	0.26	0.59	0.00	1.00	−0.33	−0.59
DD large	518	−1.66	0.36	−1.86	−1.99	−0.59	1.29	0.66
CFT speed	522	55.11	8.94	54.33	16.33	92.67	0.30	2.67
GLIAT learning	547	0.02	0.17	0.02	−0.83	0.88	0.28	4.87
GLIAT transfer	547	0.54	0.26	0.54	0.00	1.00	−0.11	−0.68
EI context	520	73.06	36.81	80.00	−102.00	149.00	−1.83	5.18
MF inverted	491	32.38	21.76	30.23	0.00	93.02	0.61	−0.34
MF upright	489	78.14	13.04	81.40	27.91	100.00	−0.94	0.97

*Note.* The NAPLS-RC was only calculated for CHR-P participants, given that its original validation was based on use in this population only. The PRL WSR and DD large were logarithmically transformed, consistent with previous work (see supplement). Descriptive statistics for each group are provided in Supplemental Table 1.

**Table 3.** Model fit and comparisons

Model	AIC	LL	Wald *p*-value
(a) Intercept	1604	−799	
(b) Demographic	1620	−783	(a) vs. (b), *p* = .39
(c) Task Battery	1578	−735	(a) vs. (c), *p*<.001

*Note.* Model fit indices and tests are based on pooled comparisons across 100 imputed datasets.

**Table 4.** Significant predictors from the task battery multinomial regression

Predictor	Estimate	SE	Z	*p* value	OR
*HC vs. CHR-P (SN = .87, SP = .38, PPV = .72, NPV = .61)*					
KB Control	−0.27	0.12	−2.20	.029	0.76
PRL WSR	0.34	0.13	2.57	.010	1.41
SWS c	0.28	0.14	2.05	.041	1.33
CFT speed	0.25	0.13	2.00	.046	1.29
MF inverted	−0.61	0.18	−3.33	.001	0.54

*CLN vs. CHR-P (SN = .87, SP = .43, PPV = .84, NPV = .50)*					
MF inverted	−0.71	0.22	−3.20	.001	0.49
	
*PLE vs. CHR-P (SN = .87, SP = .21, PPV = .71, NPV = .42)*					
EI context	−0.28	0.12	−2.31	.019	0.75
MF inverted	−0.52	0.18	−2.80	.005	0.59

*Note.* All predictors were standardized and keyed in the theorized direction of pathology. Odds ratio (OR) values > 1.00 indicate a higher likelihood of being CHR-P. SN = Sensitivity, SP = Specificity, PPV = Positive Predictive Value, NPV = Negative Predictive Value.

## Footnotes

1. Zero-order correlations are presented in the supplement to the article (Supplemental Tables 2 and 3).

2. A follow-up analysis removing MF variables from the model indicated that the model remained significantly predictive of group status, though was less accurate (see Supplemental Results).

## References

[1] Charlson FJ, Ferrari AJ, Santomauro DF, Diminic S, Stockings E, Scott JG, Global Epidemiology and Burden of Schizophrenia: Findings From the Global Burden of Disease Study 2016. Schizophr Bull. 2018;44:1195–1203. 29762765 10.1093/schbul/sby058 PMC6192504

[2] Fusar-Poli P, Borgwardt S, Bechdolf A, Addington J, Riecher-Rössler A, Schultze-Lutter F, The Psychosis High-Risk State: A Comprehensive State-of-the-Art Review. JAMA Psychiatry. 2013;70:107. 23165428 10.1001/jamapsychiatry.2013.269 PMC4356506

[3] De Pablo GS, Radua J, Pereira J, Bonoldi I, Arienti V, Besana F, Probability of Transition to Psychosis in Individuals at Clinical High Risk: An Updated Meta-analysis. JAMA Psychiatry. 2021;78:970–978. 34259821 10.1001/jamapsychiatry.2021.0830 PMC8281006

[4] Addington J, Stowkowy J, Liu L, Cadenhead KS, Cannon TD, Cornblatt BA, Clinical and functional characteristics of youth at clinical high-risk for psychosis who do not transition to psychosis. Psychol Med. 2019;49:1670–1677. 30176955 10.1017/S0033291718002258

[5] McGlashan T, Walsh B, Woods S. The Psychosis-Risk Syndrome: Handbook for Diagnosis and Follow-Up. Oxford University Press, USA; 2010.

[6] Subotnik KL, Nuechterlein KH, Irzhevsky V, Kitchen CM, Woo SM, Mintz J. Is unawareness of psychotic disorder a neurocognitive or psychological defensiveness problem? Schizophr Res. 2005;75:147–157. 15885506 10.1016/j.schres.2004.12.005

[7] Gold JM, Corlett PR, Strauss GP, Schiffman J, Ellman LM, Walker EF, Enhancing Psychosis Risk Prediction Through Computational Cognitive Neuroscience. Schizophr Bull. 2020:sbaa091. 10.1093/schbul/sbaa091 PMC7707066 32648913

[8] Corlett PR, Fletcher PC. The neurobiology of schizotypy: Fronto-striatal prediction error signal correlates with delusion-like beliefs in healthy people. Neuropsychologia. 2012;50:3612–3620. 23079501 10.1016/j.neuropsychologia.2012.09.045 PMC3694307

[9] Suthaharan P, Reed EJ, Leptourgos P, Kenney JG, Uddenberg S, Mathys CD, Paranoia and belief updating during the COVID-19 crisis. Nat Hum Behav. 2021;5:1190–1202. 34316049 10.1038/s41562-021-01176-8 PMC8458246

[10] Powers AR, Mathys C, Corlett PR. Pavlovian conditioning-induced hallucinations result from overweighting of perceptual priors. Science. 2017;357:596–600. 28798131 10.1126/science.aan3458 PMC5802347

[11] Gold JM, Strauss GP, Waltz JA, Robinson BM, Brown JK, Frank MJ. Negative symptoms of schizophrenia are associated with abnormal effort-cost computations. Biol Psychiatry. 2013;74:130–136. 23394903 10.1016/j.biopsych.2012.12.022 PMC3703817

[12] Heerey EA, Robinson BM, McMahon RP, Gold JM. Delay discounting in schizophrenia. Cognit Neuropsychiatry. 2007;12:213–221. 17453902 10.1080/13546800601005900 PMC3746343

[13] Silverstein SM, Keane BP. Perceptual Organization Impairment in Schizophrenia and Associated Brain Mechanisms: Review of Research from 2005 to 2010. Schizophr Bull. 2011;37:690–699. 21700589 10.1093/schbul/sbr052 PMC3122298

[14] Millman ZB, Gold JM, Mittal VA, Schiffman J. The Critical Need for Help-Seeking Controls in Clinical High-Risk Research. Clin Psychol Sci. 2019;7:1171–1189. 33614257 10.1177/2167702619855660 PMC7891463

[15] Schiffman J, Ellman LM, Mittal VA. Individual Differences and Psychosis-Risk Screening: Practical Suggestions to Improve the Scope and Quality of Early Identification. Front Psychiatry. 2019;10:6. 30837898 10.3389/fpsyt.2019.00006 PMC6382740

[16] Mittal VA, Ellman LM, Strauss GP, Walker EF, Corlett PR, Schiffman J, Computerized Assessment of Psychosis Risk. J Psychiatry Brain Sci. 2021;6:e210011. 10.20900/jpbs.20210011 PMC8302046 34307899

[17] Addington J, Liu L, Brummitt K, Bearden CE, Cadenhead KS, Cornblatt BA, North American Prodrome Longitudinal Study (NAPLS 3): Methods and baseline description. Schizophr Res. 2020. 18 April 2020. 10.1016/j.schres.2020.04.010. PMC7572535 32317224

[18] First MB, Williams JB, Karg RS, Spitzer RL. Structured clinical interview for DSM-5—Research version (SCID-5 for DSM-5, research version; SCID-5-RV). Arlington, VA: American Psychiatric Association; 2015.

[19] Cannon TD, Yu C, Addington J, Bearden CE, Cadenhead KS, Cornblatt BA, An Individualized Risk Calculator for Research in Prodromal Psychosis. Am J Psychiatry. 2016;173:980–988. 27363508 10.1176/appi.ajp.2016.15070890 PMC5048498

[20] Keefe RSE, Goldberg TE, Harvey PD, Gold JM, Poe MP, Coughenour L. The Brief Assessment of Cognition in Schizophrenia: reliability, sensitivity, and comparison with a standard neurocognitive battery. Schizophr Res. 2004;68:283–297. 15099610 10.1016/j.schres.2003.09.011

[21] Brandt J, Benedict RH. Hopkins verbal learning test–revised: professional manual. Psychological Assessment Resources; 2001.

[22] Dohrenwend BS, Askenasy AR, Krasnoff L, Dohrenwend BP. Exemplification of a Method for Scaling Life Events: The PERI Life Events Scale. J Health Soc Behav. 1978;19:205–229. 681735

[23] Carrión RE, Auther AM, McLaughlin D, Olsen R, Addington J, Bearden CE, The Global Functioning: Social and Role Scales—Further Validation in a Large Sample of Adolescents and Young Adults at Clinical High Risk for Psychosis. Schizophr Bull. 2019;45:763–772. 30351423 10.1093/schbul/sby126 PMC6581127

[24] Alderson-Day B, Lima CF, Evans S, Krishnan S, Shanmugalingam P, Fernyhough C, Distinct processing of ambiguous speech in people with non-clinical auditory verbal hallucinations. Brain. 2017;140:2475–2489. 29050393 10.1093/brain/awx206

[25] Treadway MT, Buckholtz JW, Schwartzman AN, Lambert WE, Zald DH. Worth the ‘EEfRT’? The effort expenditure for rewards task as an objective measure of motivation and anhedonia. PloS One. 2009;4:e6598. 19672310 10.1371/journal.pone.0006598 PMC2720457

[26] Strauss GP, Ruiz I, Visser KH, Crespo LP, Dickinson EK. Diminished Hedonic response in neuroleptic-free youth at ultra high-risk for psychosis. Schizophr Res Cogn. 2018;12:1–7. 29928593 10.1016/j.scog.2017.12.001 PMC6006907

[27] Damme KSF, Osborne KJ, Gold JM, Mittal VA. Detecting motor slowing in clinical high risk for psychosis in a computerized finger tapping model. Eur Arch Psychiatry Clin Neurosci. 2020;270:393–397. 31432263 10.1007/s00406-019-01059-0 PMC7031007

[28] Mittal VA, Gupta T, Keane BP, Silverstein SM. Visual context processing dysfunctions in youth at high risk for psychosis: Resistance to the Ebbinghaus illusion and its symptom and social and role functioning correlates. J Abnorm Psychol. 2015;124:953–960. 26237183 10.1037/abn0000082 PMC4658222

[29] Silverstein SM, Thompson JL, Gold JM, Schiffman J, Waltz JA, Williams TF, Increased face detection responses on the mooney faces test in people at clinical high risk for psychosis. Npj Schizophr. 2021;7:1–7. 34001909 10.1038/s41537-021-00156-1 PMC8129098

[30] Team RStudio. RStudio. 2020.

[31] van Buuren S, Groothuis-Oudshoorn K. mice: Multivariate Imputation by Chained Equations in R. J Stat Softw. 2011;045.

[32] Collin G, Nieto-Castanon A, Shenton ME, Pasternak O, Kelly S, Keshavan MS, Brain functional connectivity data enhance prediction of clinical outcome in youth at risk for psychosis. Neurolmage Clin. 2020;26:102108. 10.1016/j.nicl.2019.102108 PMC7229353 31791912

[33] Sanfelici R, Dwyer DB, Antonucci LA, Koutsouleris N. Individualized Diagnostic and Prognostic Models for Patients With Psychosis Risk Syndromes: A Meta-analytic View on the State of the Art. Biol Psychiatry. 2020;88:349–360. 32305218 10.1016/j.biopsych.2020.02.009

[34] Woods SW, Parker S, Kerr MJ, Walsh BC, Wijtenburg SA, Prunier N, Development of the PSYCHS: Positive SYmptoms and Diagnostic Criteria for the CAARMS Harmonized with the SIPS. Early Interv Psychiatry. in press. in press. 10.1111/eip.13457. PMC10899527 37641537

[35] Mueser KT, DeTore NR, Kredlow MA, Bourgeois ML, Penn DL, Hintz K. Clinical and demographic correlates of stigma in first-episode psychosis: the impact of duration of untreated psychosis. Acta Psychiatr Scand. 2020;141:157–166. 31557309 10.1111/acps.13102 PMC6980158

[36] Kular A, Perry BI, Brown L, Gajwani R, Jasini R, Islam Z, Stigma and access to care in first-episode psychosis. Early Interv Psychiatry. 2019;13:1208–1213. 30411522 10.1111/eip.12756

[37] Vega WA, Lewis-Fernández R. Ethnicity and variability of psychotic symptoms. Curr Psychiatry Rep. 2008;10:223–228. 18652790 10.1007/s11920-008-0037-y

[38] Mirza A, Birtel MD, Pyle M, Morrison AP. Cultural Differences in Psychosis: The Role of Causal Beliefs and Stigma in White British and South Asians. J Cross-Cult Psychol. 2019;50:441–459.

[39] Huys QJM, Maia TV, Frank MJ. Computational psychiatry as a bridge from neuroscience to clinical applications. Nat Neurosci. 2016;19:404–413. 26906507 10.1038/nn.4238 PMC5443409

[40] Corlett PR, Fletcher PC. Computational psychiatry: a Rosetta Stone linking the brain to mental illness. Lancet Psychiatry. 2014;1:399–402. 26361002 10.1016/S2215-0366(14)70298-6

